# Comparability of a Blood-Pressure-Monitoring Smartphone Application with Conventional Measurements—A Pilot Study

**DOI:** 10.3390/diagnostics12030749

**Published:** 2022-03-19

**Authors:** Annina S. Vischer, Jana Rosania, Thenral Socrates, Christina Blaschke, Jens Eckstein, Yara-Maria Proust, Guillaume Bonnier, Martin Proença, Mathieu Lemay, Thilo Burkard

**Affiliations:** 1 Medical Outpatient Department and Hypertension Clinic, ESH Hypertension Centre of Excellence, University Hospital Basel, 4031 Basel, Switzerland; jana.rosania@stud.unibas.ch (J.R.); thenral.socrates@usb.ch (T.S.); christina.blaschke@usb.ch (C.B.); thilo.burkard@usb.ch (T.B.); 2 Department of Internal Medicine, University Hospital Basel, 4031 Basel, Switzerland; jens.eckstein@usb.ch; 3 Centre Suisse d’Electronique et de Microtechnique (CSEM), 2002 Neuchatel, Switzerland; yara-maria.proust@csem.ch (Y.-M.P.); guillaume.bonnier@csem.ch (G.B.); martin.proenca@csem.ch (M.P.); mathieu.lemay@csem.ch (M.L.); 4 Department of Cardiology, University Hospital Basel, 4031 Basel, Switzerland

**Keywords:** blood-pressure measurement, arterial hypertension, smartphone application, new technologies, diagnostic techniques, monitoring, blood-pressure monitoring

## Abstract

(1) Background: New cuffless technologies attempting blood-pressure measurements (BPM) offer possibilities to improve hypertension awareness and control. The aim of this study was to compare a smartphone application (app)-based algorithm with office BPM (OBPM). (2) Methods: We included consecutive patients with an indication for ambulatory BPM. The smartphone app (RIVA digital) acquired the pulse wave in the fingers’ arterial bed using the phone’s camera and estimated BP based on photoplethysmographic (PPG) waveforms. Measurements were alternatingly taken with an oscillometric cuff-based device and smartphone BPM (AppBP) on two consecutive days. AppBP were calibrated to the first OBPM. Each AppBP was compared to its CuffBP (mean of the previous/following OBPM). (3) Results: 50 participants were included, resulting in 50 AppBP values on Day 1 and 33 on Day 2 after exclusion of 225 AppBP due to insufficient quality. The mean ± SD of the differences between AppBP and CuffBP was 0.7 ± 9.4/1.0 ± 4.5 mmHg (*p*-value 0.739/0.201) on Day 1 and 2.6 ± 8.2/1.3 ± 4.1 mmHg (*p*-value 0.106/0.091) on Day 2 for systolic/diastolic values, respectively. There were no significant differences between the deviations on Day 1 and Day 2 (*p*-value 0.297/0.533 for systolic/diastolic values). Overall, there were 10 (12%) systolic measurement pairs differing by >15 mmHg. (4) Conclusions: In this pilot evaluation, the RIVA Digital app shows promising results when compared to oscillometric cuff-based measurements, especially regarding diastolic values. Its differences between AppBP–CuffBP have a good stability one day after calibration. Before clinical use, signal acquisition needs improvement and the algorithm needs to undergo formal validation against a gold-standard BPM method.

## 1. Introduction

Arterial hypertension (AHT) is the most important preventable risk factor for cardiovascular disease (CVD), all-cause mortality and morbidity worldwide [1,2]. Despite the still high prevalence of AHT, there are encouraging improvements in awareness and treatment of AHT in both sexes, but especially in women [3]. Nevertheless, only between a third and a half of those with AHT are aware of their condition or have their blood pressure (BP) adequately controlled [4,5]. Therefore, continuous improvement in the diagnosis and treatment of AHT remains important.

To date, the following methods are used to diagnose AHT: conventional office blood pressure measurement (OBPM), unattended automated OBPM, and out-of-office blood pressure measurements [6]. Out-of-office blood pressure measurements refer to the use of either home blood-pressure monitoring (HBPM) or ambulatory 24 h blood-pressure measurement (ABPM), the latter usually over 24 h [6]. An advantage of both measurement methods is that they can detect masked and white-coat hypertension, and therefore prevent over- and under-treatment, whereas the reported discomfort with both methods may be a disadvantage [6,7]. Nevertheless, HBPM has the potential to improve BP control by providing simple and patient-friendly solutions [8].

As information technology and the use of smartphones continues to grow, so do the opportunities to find new ways to measure BP, such as smartphone-based applications (apps). The use of smartphones has increased massively over recent years. It is estimated that over 6.8 billion mobile phones will be used worldwide by 2022 [9]. In the context of HBPM, smartphone technology is used with contrasted success to provide more effective and comfortable methods. However, this is mainly (70%) for documentation purposes of HBPM, and only less than 2% of apps claim to measure BP [10]. There are different technologies involving smartphones claiming to be able to estimate BP. Most commonly, this is undertaken by using the in-built smartphone camera to assess photoplethysmographic (PPG) recordings of pulse wave [11]. Other technologies, for example, involve the combination of PPG with smartphone-case-based single-channel ECG [12], an oscillometric finger-pressing method [13], the combination of heart rate and modified normalized pulse volume based on PPG [14] or assessment of facial blood-flow data [15]. The app tested in this study is the RIVA Digital Blood Pressure app using a PPG technology, which was created to raise awareness of AHT and the importance of BP monitoring among the Swiss population, and to incentivize people to achieve and maintain a healthy lifestyle [16]. Studies testing PPG-based apps have shown mixed results [17]. There are apps which clearly fail validation, such as the InstantBP app [18] or the app analysed in the iPARR trial [11,19]. A recent systematic review on smartphone and smartwatch BPM states that technology is clearly improving, and opens a possibility to enable valid BPM using smartphone technology [20]. They have found a single app passing AAMI/ESH/ISO validation, which applies transdermal optical imaging (TOI) instead of PPG technology [15]. Since the publication of this review, other apps have been released and tested which show promising results, such as the OptiBP app, which uses the same algorithm as the app tested in our study, but uses different pre-processing [21,22].

The aim of this study was to compare BP values determined by this RIVA Digital smartphone app-based HBPM method with conventional oscillometric cuff-based OBPM.

## 2. Materials and Methods

For this pilot study, BP values estimated by the RIVA Digital Blood Pressure app were compared against conventional cuff-based OBPM. The app acquires the pulse wave in the finger’s arterial bed using the phone’s camera and estimates BP based on the analysis of the PPG waveform. 

### 2.1. Patient Selection

Consecutive patients older than 18 years of age and referred for an ambulatory blood-pressure measurement (ABPM) at the Medical Outpatient Department at the University Hospital Basel were able to participate in this study. Patients with missing informed consent or physical conditions precluding BP measurements such as missing arms were excluded from the project. 

### 2.2. Study Procedure

The participants were present on two consecutive days. The study measurements were taken before and after the ABPM, the latter being not part of the analysis. After fitting of the cuff-based BPM device and the smartphone, the participants were sitting quietly, and relaxed and rested for 3–5 min before the measurements started [6]. No conversations were allowed with the participant during the procedure [6].

**Figure 1:**
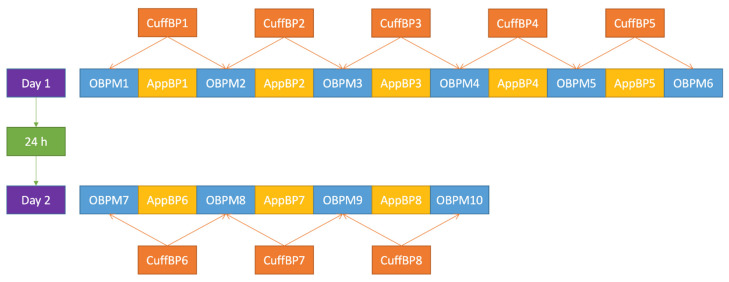
Blood-pressure measurement procedure applied in this trial. OBPM: (oscillometric) office blood-pressure measurement; AppBP: app-based blood-pressure measurement; CuffBP: cuff-based blood-pressure measurement: mean between two adjacent OBPM.

BP was measured every minute, alternating between the two methods, and starting with cuff-based BPM (OBPM). Six OBPM and five app measurements (AppBP) were taken on the first day (Day 1). To evaluate the stability of the calibration over time, on the second day (Day 2), four OBPM and three AppBP measurements were taken (Figure 1). 

#### 2.2.1. Conventional Measurement, OBPM

For the measurements with the conventional device, later referred to as OBPM, a conventional (cuff-based) BP monitor (Welch Allyn Connex^®^ Spot Monitor, with the SureBP^®^ algorithm) was used. This device differed in mean ± standard deviation (SD) by −2.15 ± 7.44 mmHg (systolic) and −0.55 ± 5.98 mmHg (diastolic) from auscultation BPM during its validation study [23]. The correct cuff size for each arm size was used, as measured with a measuring tape [6]. The fitted BP cuff was placed on the upper arm on the non-dominant side. The operator remained in the room for all measurements. 

#### 2.2.2. App-Based Measurements, AppBP

The Riva Digital app was installed on six different iPhone 6S (Apple) phones provided by Centre Suisse d’Electronique et de Microtechnique (CSEM). Every iPhone had the same iOs version (version 15.1). The Riva Digital app differed from the publicly available version, as it did not show any BP values, to keep the study staff blinded. The working principle of the app’s BP measurement algorithm is detailed in Section 2.2.5.

For the measurements with the app, the smartphone was placed next to the participant with the screen facing the table, so that they could place their finger on the camera (Supplemental Figure S1). Either the index, the middle, or the ring finger was used depending on which was most comfortable for the participant. However, the same finger was used consistently throughout the measurements. The non-dominant arm was used, thus the same arm as where the cuff was attached. The settings on the app were handled by the study staff. This ensured that the participants only had to lift their finger and place it on the camera and flashlight of the smartphone, allowing them to remain calmly seated with the arm placed in the same relaxed position on the table during all alternating OBPM and app measurements.

#### 2.2.3. Merging of the Measurements

All participants were given a unique identification number, which was automatically registered by the app. This number was sent to CSEM to identify study participants. The acquired PPG signals first underwent a quality check (see details in Section 2.2.5), and recordings which did not meet the quality requirements were excluded. All other PPG signals, “uncalibrated AppBP values” were computed by CSEM (see details in Section 2.2.5). These values were then sent to the study staff at the University Hospital Basel, where the received uncalibrated AppBP values were added to the database for each participant. The study staff at CSEM were blinded to the OBPM. 

In a second step, the study staff at the University Hospital Basel sent the first OBPM measurement (OBPM1 in Figure 1) to the study staff at CSEM, which was then used to calibrate the AppBP estimates for each patient (see details in Section 2.2.5); the latter were referred to as “calibrated AppBP values” and added to the database of the University Hospital Basel. The CSEM staff remained blinded for all other OBPM measurements. 

#### 2.2.4. Cuff-Derived Values, CuffBP

For each AppBP value, the cuff-derived value used for comparison was the mean value—hereafter referred to as “CuffBP”—of each adjacent OBPM, that is the OBPM taken before and after the AppBP value (see also Figure 1).

#### 2.2.5. Working Principle of the App

In order to estimate a BP value, as a first step, the app averages the pixels from the green channel of the central region of each image in the video sequence recorded by the smartphone camera to obtain a PPG signal. Each pulse in the PPG signal is given a quality index according to its similarity to its neighbouring pulses, resulting in less weight given to pulses with aberrant morphologies. All pulses are then averaged to obtain a representative pulse wave shape. An acceptance criterion based on the average quality of the pulses and the waveform of the averaged pulse is computed, which allows for automatic rejection of average pulses with un-physiological morphology or those obtained from PPG signals with low-quality pulses (e.g., in cases of excessive finger movement, poor positioning or excessive finger–camera pressure). The acceptance threshold on the quality index was derived empirically from previous studies as a trade-off between data acceptance rate and BP accuracy. Each accepted average waveform is analysed by the algorithm [24], which characterises the morphological variations of the pulse at various time resolutions from derivative-based features. The features are mapped to uncalibrated BP values through a dedicated model which has been previously trained on a different dataset [25]. The BP values calculated by the algorithm are uncalibrated (hereafter, “uncalibrated AppBP values”) in the sense that the algorithm arbitrarily assumes the initial BP of the user to be 120/80 mmHg, and then calculates all following BP measurements as variations around that initial measurement. Therefore, the app is designed in such a way that the user can input his/her own true initial BP instead of using the default 120/80 mmHg values. In that case, these BP measurements are referred to as “calibrated AppBP values”.

### 2.3. Statistical Analyses

All calculations were performed separately for systolic and diastolic values. Continuous data were reported as “mean ± standard deviation” (SD) or as “median (inter-quartile range (IQR))”, as applicable. We used the Shapiro–Wilk Test to test for normality. Each AppBP value was compared to its corresponding CuffBP by calculating the difference as AppBP–CuffBP. We tested for association using the related-samples Wilcoxon signed rank-test and Bland–Altman plots, which were designed according to the requirements of the AAMI/ESH/ISO validation protocol [26]. All calculations were performed over all measurements, as well as on a participant level, when we started by calculating the mean difference for each participant and continued using the results per participant. The differences between Day 1 and Day 2 were calculated using the Mann–Whitney-U test.

All statistical analyses were performed using SPSS version 22 and R version 3.6.0 [27]. 

#### Comparison Based on Standards

There is currently no internationally accepted standard for evaluating the accuracy and precision of cuffless BP monitors. Moreover, existing standards for cuff-based devices, such as the European Society of Hypertension International Protocol for the validation of blood pressure measuring devices in adults, published in 2010 (ESH-IP 2010) [28], or the Universal Standard for the validation of blood pressure measuring devices (AAMI/ESH/ISO) [26,29], require auscultatory measurements as a gold-standard reference for determining the accuracy and precision of the device under test. For these reasons, in the present study, these standards can only be taken as points of comparison but cannot be formally applied. This study is—before anything—a method-comparison pilot study, and is not by any means a validation study. Nevertheless, for research purposes, we will try to apply these standards as faithfully as possible in our analysis, by considering the oscillometric cuff-based BP measurements of a formally validated device (CuffBP) as gold-standard reference values, and the app-based measurements (AppBP) as those of the device under test.

The ESH-IP 2010 requests the inclusion of 33 participants with 99 measurement pairs [28]. A device will fail the ESH-IP 2010 if more measurement pairs differ by more than 5, 10, or 15 mmHg, respectively, than the number stated in Table 1 [28,30]. For example, if a test device delivers four measurements with a difference of >15 mmHg and 13 measurements with a difference of >10 mmHg to the CuffBP value, it cannot pass the ESH-IP 2010 validation criteria. In addition, if, for example, the test device delivers seven measurements with a difference of >15 mmHg to the CuffBP, it cannot pass the ESH-IP 2010 validation criteria. The AAMI/ESH/ISO standard published in 2018 requests the inclusion of at least 85 participants and at least 255 measurement pairs with (a) a cohort-wise mean difference (bias) within ±5 mmHg and an SD of the differences of ≤8 mmHg over all measurement pairs, and (b) an SD of the per-participant averages of the pairs of measurements smaller than a given threshold [26,29]. The threshold for this SD value depends on the cohort-wise bias, and is, for example, 4.79 mmHg for a bias of 5 mmHg [26,29]. As stated earlier, it is important to remember that these figures and limits only apply when the device under test (AppBP in our study) is compared to an accepted BP reference device (auscultatory method measurement), which it is not in this pilot study. Thus, the (AppBP–CuffBP) differences reflect the disagreement between both devices rather than the inaccuracy and imprecision of AppBP only, as they are also affected by the oscillometric cuff’s own inaccuracy and imprecision. 

Finally, as less than the required number of participants were included in our pilot study according to the AAMI/ESH/ISO protocol, we estimate a “best-case scenario SD” to predict the possibility of passing the AAMI/ESH/ISO protocol, where CuffBP would be considered a gold-standard measurement. The SD is the square root of the variance, which is the sum of the squared differences of each data point (i.e., each difference AppBP–CuffBP) with the cohort-wise bias (mean of all differences AppBP–CuffBP) [31]. We modelled a best-case scenario SD by calculating the SD over all pairs of measurement from our results (Criterion 1 for individual BP readings [29]) for different assumed biases between −5 and 5 mmHg, assuming that all missing measurements to reach 255 pairs of measurements would be equal to the assumed mean [30]. Similarly, we proceeded with the mean of all measurement pairs of each participant (Criterion 2 for individual subjects [29]) but used assumed missing values to reach 85 participants [30]. The results were displayed graphically by drawing the maximally allowed SD and the calculated best-case scenario SD from the AppBP–CuffBP differences for each assumed mean. 

### 2.4. Ethics

The local ethics committee (Ethikkommission Nordwest- und Zentralschweiz/Ethics Committee Northwestern and Central Switzerland, Basel, Switzerland, EKNZ, EKNZ2020-00009) approved this trial. This trial was registered on ClinicalTrials.gov (NCT04461834) and was compliant with the Declaration of Helsinki. All patients gave informed written consent. 

## 3. Results

### 3.1. Baseline Characteristics

In total, 50 consecutive participants were included in this trial, of which 24 were women (48.0%). Furthermore, 44 participants (88.0%) were measured on the left-hand side, 42 (84.0%) were measured at the second (index) finger, and 8 (16.0%) at the third (middle) finger. In addition, 38 participants (76.0%) had known AHT and 30 (60.0%) were receiving antihypertensive treatment. This resulted in 400 valid CuffBP values. The post-recording quality threshold was not met in 225 app-based measurements, which had to be excluded. This left us with 175 uncalibrated values and 83 calibrated values. 

Systolic BP ranged from 89 to 202 mmHg for all 400 CuffBP values, with a mean of 126 ± 16 mmHg. Diastolic BP ranged from 48 to 96 mmHg, with a mean of 79 ± 8.7 mmHg. Furthermore, 17 (4.25%) systolic CuffBP values were ≤100 mmHg, 59 (14.75%) were ≥140 mmHg, and 11 (2.75%) were ≥160 mmHg [29]. Additionally, 11 (2.8 %) diastolic CuffBP values were ≤60 mmHg, 110 (27.5%) were ≥85 mmHg, but none were ≥100 mmHg [29]. 

### 3.2. Results for Uncalibrated AppBP Values

Due to insufficient signal quality, 225 (56.25%) uncalibrated AppBP values had to be excluded, resulting in 175 valid uncalibrated AppBP values from 43 (86.0%) participants. Furthermore, 6 participants (12.0%) had one valid AppBP value, 7 (14.0%) had two, 3 (6.0%) had three, 6 (12.0%) had four, 10 (20.0%) had five, 6 (12.0%) had six, 4 (8.0%) had seven, 1 (2.0%) had eight, and 7 (14.0%) had no valid uncalibrated AppBP values over the 2 days of measurement. 

Systolic uncalibrated AppBP values (median 120.0 (IQR 117.0–122.4) mmHg) were significantly lower than the corresponding systolic CuffBP values (median 126.0 (IQR 116.5–133.0) mmHg, *p*-value < 0.0005, z-value −5.325). Diastolic uncalibrated AppBP values (median 80.0 (IQR 78.8–81.1) mmHg) did not significantly differ from the corresponding diastolic CuffBP values (median 79.5 (IQR 74.0–85.5) mmHg, *p*-value 0.316, z-value 1.002). 

**Figure 2:**
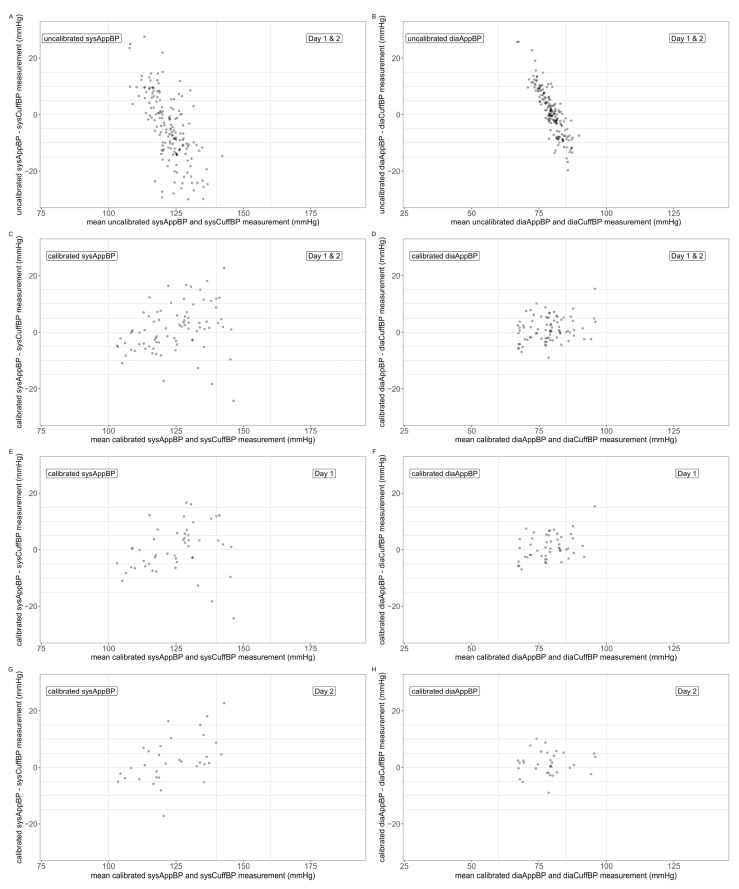
Bland–Altman plots comparing the mean of AppBP and CuffBP (x-axis) with the difference AppBP–CuffBP (y-axis). Uncalibrated AppBP Day 1 and Day 2: panels (**A**,**B**); calibrated AppBP, Day 1 and Day 2: panels (**C**,**D**); calibrated AppBP, Day 1: panels (**E**,**F**); calibrated AppBP, Day 2: panels (**G**,**H**). Systolic values: panels (**A**,**C**,**E**,**G**); diastolic values: panels (**B**,**D**,**F**,**H**).

Mean, SD and range of the differences between the uncalibrated AppBP and the CuffBP can be found in Table 2, as well as deviation categories for the absolute differences. Bland–Altman plots are depicted in Figure 2, panels (A) and (B), showing an overestimation of lower BP and underestimation of higher BP values.

### 3.3. Results for Calibrated AppBP Values

#### 3.3.1. Day 1 and Day 2 Combined

Only AppBP sets with a valid first AppBP measurement could be included, as it is the one used for calibration. After calibration, the first measurement had to be excluded, resulting in 83 calibrated AppBP values from 19 (38.0%) participants. No participant had only 1 calibrated AppBP value, 2 participants (4.0%) had two, 3 (6.0%) had three, 5 (10.0%) had four, 5 (10.0%) had five, 3 (6.0%) had six, 1 (2.0%) had seven, and 31 (62.0%) had no calibrated AppBP values.

Systolic calibrated AppBP values (median 127.6 (IQR 113.8–135.3) mmHg) did not significantly differ from the corresponding systolic CuffBP values (median 125.0 (IQR 115.0–132.0) mmHg, *p*-value 0.234, z-value 1.190). Diastolic calibrated AppBP values (median 79.3 (IQR 74.0–83.6) mmHg) were significantly higher than the corresponding diastolic CuffBP values (78.5 (IQR 72.0–82.5) mmHg, *p*-value 0.041, z-value 2.039).

Mean, SD and range of the differences between the calibrated AppBP and the CuffBP can be found in Table 2, as well as deviation categories for the absolute differences. Bland–Altman plots are depicted in Figure 2, panels (C) and (D).

#### 3.3.2. Day 1

From Day 1, 50 calibrated AppBP values from 19 (38.0%) participants were available. Overall, 2 participants (4.0%) had only 1 calibrated AppBP value on Day 1, 7 participants (14.0%) had two, 6 (12.0%) had three, 4 (8.0%) had four, and 31 (62.0%) had no calibrated AppBP values on Day 1.

Systolic calibrated AppBP values (median 128.8 (IQR 113.5–134.5) mmHg) did not significantly differ from the corresponding systolic CuffBP values (median 125.5 (IQR 116.1–132.1) mmHg, *p*-value 0.739, z-value 0.333). Diastolic calibrated AppBP values (median 80.0 (IQR 74.1–84.2) mmHg) did not significantly differ from the corresponding diastolic CuffBP values (median 78.8 (IQR 72.4–83.0) mmHg, *p*-value 0.201, z-value 1.279).

Mean, SD and range of the differences between the calibrated AppBP and the CuffBP for Day 1 can be found in Table 3, as well as deviation categories for the absolute differences. Bland–Altman plots are depicted in Figure 2, panels (E) and (F), showing an improved systematic error with less overestimation of low and underestimation of high BP values.

#### 3.3.3. Day 2

From Day 2, 33 calibrated AppBP values from 15 participants (30.0%) were available. Overall, 3 participants (6.0%) had only 1 calibrated AppBP value on Day 2, 6 participants (12.0%) had two, 6 (12.0%) had three, and 35 (70.0%) had no calibrated AppBP values on Day 2.

Systolic calibrated AppBP values (median 123.0 (IQR 114.6–137.1) mmHg) did not significantly differ from the corresponding systolic CuffBP values (median 120.5 (IQR 113.8–132.0) mmHg, *p*-value 0.106, z-value 1.617). Diastolic calibrated AppBP values (median 79.1 (IQR 73.8–82.2) mmHg) did not significantly differ from the corresponding diastolic CuffBP values (median 78.5 (IQR 70.8–81.5) mmHg, *p*-value 0.091, z-value 1.689).

Mean, SD and range of the differences between the calibrated AppBP and the CuffBP for Day 2 can be found in Table 3, as well as deviation categories for the absolute differences. Bland–Altman plots are depicted in Figure 2, panels (G) and (H).

The differences between the calibrated AppBP and the CuffBP did not differ significantly between Day 1 and Day 2 for systolic (*p*-value 0.297) and diastolic values (*p*-value 0.533).

#### 3.3.4. Differences at Participant Level

After calculating the mean difference between AppBP and CuffBP for each participant, we calculated the average per-participant mean, SD, minimum and maximum AppBP–CuffBP difference (Supplemental Table S1).

### 3.4. Comparison Based on Standards

#### 3.4.1. ESH-IP 2010 Validation Protocol

For the uncalibrated AppBP, there are more measurement pairs than requested by the ESH-IP 2010 validation protocol [28]. Therefore, we cannot give a definite statement on the passing or failing potential of this approach. However, with 24% AppBP values deviating >15 mmHg from the CuffBP, it is unlikely that the uncalibrated AppBP would pass the ESH-IP 2010 protocol, considering CuffBP as a valid reference.

Regarding the calibrated AppBP results combined from Day 1 and Day 2 (n = 83), we have >7 systolic AppBP measurements deviating >15 mmHg from the corresponding CuffBP value. If CuffBP was as accurate as a gold-standard reference, this would also preclude a passing result according to the ESH-IP 2010 protocol.

#### 3.4.2. AAMI/ESH/ISO Validation Protocol

**Figure 3:**
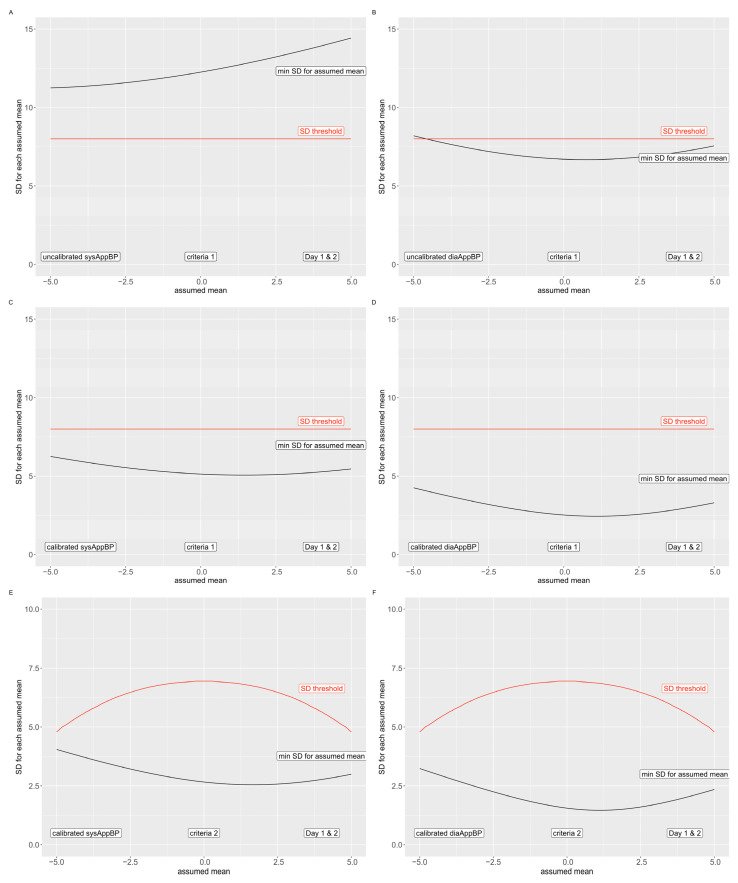
Fail criteria for the AAMI/ESH/ISO validation protocol. The red line indicates the maximal allowed SD, the black line the minimally possible SD for the included AppBP–CuffBP differences. Uncalibrated AppBP: criterion 1: panel (**A**,**B**); Calibrated AppBP: criterion 1: panel (**C**,**D**); criterion 2: panel (**E**,**F**). Systolic values: panel (**A**,**C**,**E**); diastolic values: panel (**B**,**D**,**F**).

To estimate if the AppBP app would have a potential to pass the AAMI/ESH/ISO validation protocol, we assumed that all values missing to complete the validation protocol would lead to a difference in AppBP–CuffBP which is exactly equal to the mean of all differences, and that CuffBP is as accurate as a gold-standard reference. To pass, the first criterion stated by the validation protocol is that the mean of all these differences must be between −5 and 5 mmHg, which we therefore assumed. With these assumptions, we find that all minimally reachable SD would be higher than the maximally allowed 8 mmHg (second criterion of the validation protocol) for the uncalibrated systolic AppBP (Figure 3, panel (A) and (B)). Therefore, if the CuffBP values could be considered as a valid reference, the uncalibrated AppBP version would not pass the AAMI/ESH/ISO validation protocol.

The calibrated AppBP results, however, do not surpass the maximally allowed SD of 8 mmHg using all assumptions stated above (Figure 3, panel (C) and (D)); therefore, we cannot preclude that the calibrated version of the AppBP app would pass criterion 1 of the AAMI/ESH/ISO validation protocol. In addition, regarding criterion 2 of the AAMI/ESH/ISO validation protocol, we found that the minimally possible SD by applying the assumptions above does not exceed the maximally allowed SD (Figure 3, panel (E) and (F)). Therefore, based on criterion 2, we can once again not preclude that the calibrated version of the app would pass criterion 2 of the AAMI/ESH/ISO validation protocol.

## 4. Discussion

BPM devices are usually validated according to specific protocols [26,28,29]. To date, these validation protocols are not intended for continuous or cuffless monitors [29]. Furthermore, validation studies according to recommended validation protocols are very expensive due to the high number of staff needed for every measurement (two observers and one supervisor) and the high number of patients needed to fulfil all requirements. Already, the 1990 British Hypertension Society protocol for the evaluation of automated and semi-automated BP-measuring devices have suggested the use of a stepwise approach to minimize unnecessary testing [32]. Therefore, we planned the present study to examine the AppBP as a pilot project using a similar stepwise approach.

In this pilot study, we compared a novel PPG-based BPM algorithm used in a smartphone app (RIVA Digital) as AppBP against OBPM (CuffBP) over 24 h. Our results show that PPG assessment was insufficient to meet a quality threshold in more than 50% of the participants. Using those AppBP values meeting the quality threshold, we found that, without calibration, there were differences between AppBP and CuffBP of −5.9 ± 13.6/0.7 ± 8.1 mmHg for systolic and diastolic BP values, respectively. The agreement between AppBP and CuffBP was improved (especially for systolic BP) by calibrating the AppBP values using the first OBPM value, leading to differences of 1.4 ± 8.9/1.1 ± 4.3 mmHg for systolic/diastolic values, respectively. However, there were several (AppBP-CuffBP) differences over 15 mmHg, especially for systolic values. Importantly, we found that there were no significant differences regarding the (AppBP–CuffBP) differences on the second day in comparison to the first day.

Our results suggest that this PPG-based algorithm with a single point calibration has a potential to pass the AAMI/ESH/ISO standard for BPM devices, should it in the future be approved for non-cuff-based BPM [26,29]. Indeed, while the SD of 8.9 mmHg on the systolic differences is above the threshold of 8 mmHg required by the standard, this value encompasses both the imprecision of AppBP and CuffBP in our study, as the latter is derived oscillometrically and not via the auscultatory method. The oscillometric cuff showed a disagreement of −2.15 ± 7.44 mmHg against reference auscultatory measurements in its validation study [23], and it is therefore very likely that the disagreement observed in our study between AppBP and CuffBP is at least partially imputable to the oscillometric cuff’s imprecision. The same applies regarding the observed number of AppBP–CuffBP differences over 15 mmHg, which would prevent the passing of the ESH-IP 2010 validation protocol [28], had the CuffBP values been obtained from an auscultatory reference.

Another important aspect that needs to be pointed out is the PPG data rejection rate. Considering the large number of exclusions of AppBP measurements due to insufficient PPG quality, a clear indication of a post-recording quality check is crucial. Further examination of the AppBP and potential adaptation of the algorithm is necessary, eventually according to formal validation protocols. If the app delivers consistent results, this would serve as a baseline for further research as well as allowing healthcare professionals and patients to profit from the benefits of an easy-to-apply technology. Nevertheless, the proportion of recordings with insufficient quality remains a limiting factor for its clinical use and bears the risk of misinterpretation of results. The use of more recent smartphones might help with improving the data acceptance rate as they tend to provide videos—and therefore PPG signals—with higher frame rates (which were 60 frames per second in the present study), and improved illumination (flashlight) and measurement (camera) stability and quality.

To our knowledge, this is the first study using the RIVA Digital app. In 2021, an extensive systematic review on smartphone- and smartwatch-based BPM was published, which showed impressively, that to the date of its publication, no PPG-based app has been tested which could be recommended [20]. Besides the inaccuracies detected in the app BPM, this review warned also that even wrist-worn cuff BPM devices show large inaccuracies in regular home self-measurement despite training, and that this effect could be even more pronounced in BPM taken with smartphone apps [17,20]. However, the authors state that the results are improving over time [20]. Indeed, after the publication of this review, an app called OptiBP was launched, which uses the same algorithm as the app tested in this study [21,22]. In its validation study according to the AAMI/ESH/ISO protocol, the mean difference between the OptiBP and the reference BP were 0.5 ± 7.7 mmHg for systolic and 0.4 ± 4.6 mmHg against an auscultatory method [22]. The OptiBP acceptance rate was 85% [22]. It has to be criticized though, that the protocol was not strictly followed, and many more than the 255 measurements required in the protocol were included (in some participants up to 9). This may lower the SD, which is at 7.7 mmHg for systolic values close to the failing threshold. Part of this discrepancy may be caused by their use of an auscultatory method and our use of a SmartBP technology as a comparison, and the difference in the apps using the algorithm.

Considering the high prevalence of AHT, and that a relevant proportion of people suffering from AHT are unaware of this diagnosis, easily available measurements are an important, unmet need [4,33]. However, imprecise measurements, referring to both under- and over-estimation, are associated with specific risks [33,34]. Underestimation of BP leads to missed diagnoses and therefore missed opportunities to treat AHT to reduce risk of cardiovascular events [35]. Overestimation of BP leads to false diagnoses with a potential of labelling healthy people as sick [36,37], wasting healthcare expenses on unnecessary diagnostics or treatment [38] and putting patients at risk of side-effects from medications given without indication [39]. Conventional BP-monitoring procedures such as ambulatory (ABPM) and home BP monitoring (HBPM) have their disadvantages, such as discomfort, sleep disturbance, lack of availability and high costs [7]. Devices using modern technology such as smartphone applications or wearables are generally unvalidated and unregulated [7]. Additionally, usual validation protocols are not designed and approved for the evaluation of cuffless BPM [29]. Nonetheless, smartphone-based BPM has a potential to increase availability of BPM and thus improve BP control [22].

Current cut-off BP values for the definition of hypertension are based on large meta-analyses and clinical trials including thousands of patients [6,35,40]. They are defined as values at which the benefits of treatment unequivocally outweigh the risks [6]. Since an approach including thousands of smartphone users to find the correlation between measurements and cardiovascular events is not practicable at this stage, a primary comparison with well-known measurement methods makes most sense. Many outcome studies used OBPM for their assessment [6,34]. OBPM has an independent and continuous relation to the incidence of cardiovascular endpoints such as stroke or myocardial infarction [6,35]. However, considering the correlation of OBPM with cardiovascular endpoints, adapted versions of the protocols may make sense to test for comparability of BPM to conventional OBPM, especially to discourage acceptance of low-accuracy methods.

### 4.1. Limitations

Our protocol is not suitable for validation according to international protocols and should therefore not be mistaken with a proper validation of the algorithm, though such protocols are not available for cuffless devices. Due to the low number of patients and the high number of PPG readings with insufficient quality resulting in the exclusion of a relevant number of pairs of comparisons, and even all readings in some participants, we have a limited number of comparisons. Furthermore, some participants had only one AppBP available, which limits the interpretation of the results on participant level. Furthermore, we used an oscillometric device for comparison instead of a mercury device with double stethoscope. Despite its validation for clinical use, this oscillometric device’s own inaccuracy and imprecision may at least in part contribute to the inaccuracy and imprecision attributed to the app-based BPM in the present study [23].

### 4.2. Perspectives

In summary, we show that a smartphone-based PPG algorithm provides BP values which do not differ significantly from cuff-based OBPM readings in this pilot study. This app has a potential to pass validation protocols. Smartphone-based BP measurements may in the future help to improve AHT awareness and BP control.

## 5. Conclusions

In comparison to oscillometric cuff-based measurements, the RIVA Digital smartphone app, which was calibrated to a single-point conventional BP measurement, shows promising results in this first-step pilot trial, especially regarding diastolic values. Its differences between AppBP–CuffBP have a good stability one day after calibration. Before clinical use, however, as a second step, this algorithm needs to undergo formal standardized validation against an auscultatory reference BPM method. Furthermore, PPG signal assessment needs to be improved.

## 6. Patents

M.P. and M.L. are the inventors of the algorithm tested in the present study, for which there is a patent application (WO2016138965A1).

## Figures and Tables

**Table 1:** Fail criteria if less than 33 participants with 99 measurement pairs are included. For the “two of” criterion, the test device will certainly fail if the stated number of observed biases is reached in two of the stated categories, and for the “either” criterion, if the stated number of observed biases is reached for any of the categories. Adapted after [30].

	>5 mmHg	>10 mmHg	>15 mmHg
Two of	≥27	≥13	≥4
Either	≥35	≥19	≥7

**Table 2:** Differences between uncalibrated and calibrated AppBP and CuffBP, stated as mean, standard deviation, minimum, maximum and in categories for the absolute differences.

Difference AppBP vs. CuffBD	Uncalibrated AppBP n = 175		Calibrated AppBP n = 83	
	Systolic	Diastolic	Systolic	Diastolic
Mean	−5.9	0.7	1.4	1.1
Std. deviation	13.6	8.1	8.9	4.3
Minimum	−43.8	−19.7	−24.3	−9.0
Maximum	33.8	25.8	31.0	15.33
≤5 mmHg, n (%)	40 (23.4)	81 (46.3)	42 (50.6)	60 (72.3)
≤10 mmHg, n (%)	94 (53.7)	139 (79.4)	64 (77.1)	81 (97.6)
≤15 mmHg, n (%)	133 (76.0)	167 (95.4)	73 (88.0)	82 (98.8)
>15 mmHg, n (%)	42 (24.0)	8 (4.6)	10 (12.0)	1 (1.2)

**Table 3:** Differences between calibrated AppBP and CuffBP, separated for Day 1 and Day 2, stated as mean, standard deviation, minimum, maximum and in categories for the absolute differences.

Difference	Day 1 n = 50		Day 2 n = 33	
	Systolic	Diastolic	Systolic	Diastolic
Mean	0.7	1.0	2.6	1.3
Std. deviation	9.4	4.5	8.2	4.1
Minimum	−24.3	−7.0	−17.2	−9.0
Maximum	31.0	15.3	22.7	10.1
≤5 mmHg, n (%)	24 (48.0)	36 (72.0)	18 (54.5)	24 (72.7)
≤10 mmHg, n (%)	38 (76.0)	49 (98.0)	26 (78.8)	32 (97.0)
≤15 mmHg, n (%)	45 (90.0)	49 (98.0)	28 (84.8)	33 (100)
>15 mmHg, n (%)	5 (10.0)	1 (2.0)	5 (15.2)	0 (0)

## Data Availability

The datasets generated and analyzed during the current study are not publicly available due to ethical restrictions.

## Supplementary Materials

The following supporting information can be downloaded at: https://www.mdpi.com/article/10.3390/diagnostics12030749/s1. Figure S1: Correct positioning of the patient’s finger during the measurement; Table S1: Average per-participant AppBP–CuffBP for calibrated AppBP.
