# Supplementary material for: Comparability of a Blood-Pressure-Monitoring Smartphone Application with Conventional Measurements—A Pilot Study
Source: Diagnostics (Basel). 2022 Mar 19;12(3):749. doi: 10.3390/diagnostics12030749 (PMC8947665; doi:10.3390/diagnostics12030749)
Supplement: Supplementary file 1 [file diagnostics-12-00749-s001.zip › diagnostics-1616697-supplementary.pdf]

Supplemental Table S1:

Supplemental Table S1: Average per-participant AppBP – CuffBP for calibrated AppBP.

**Mean AppBP – CuffBP**	**Day1 and Day2 n=19**		**Day1 n=19**		**Day2 n=15**	
	**Systolic**	**Diastolic**	**Systolic**	**Diastolic**	**Systolic**	**Diastolic**
Mean	1.7	1.1	1.8	1.5	2.7	1.4
Std. deviation	5.5	3.2	9.8	4.7	7.2	3.7
Minimum	-8.7	-4.7	-18.4	-6.1	-7.3	-5.4
Maximum	12.4	7.1	31.0	15.3	20.4	8.9

Supplemental Figure S1:

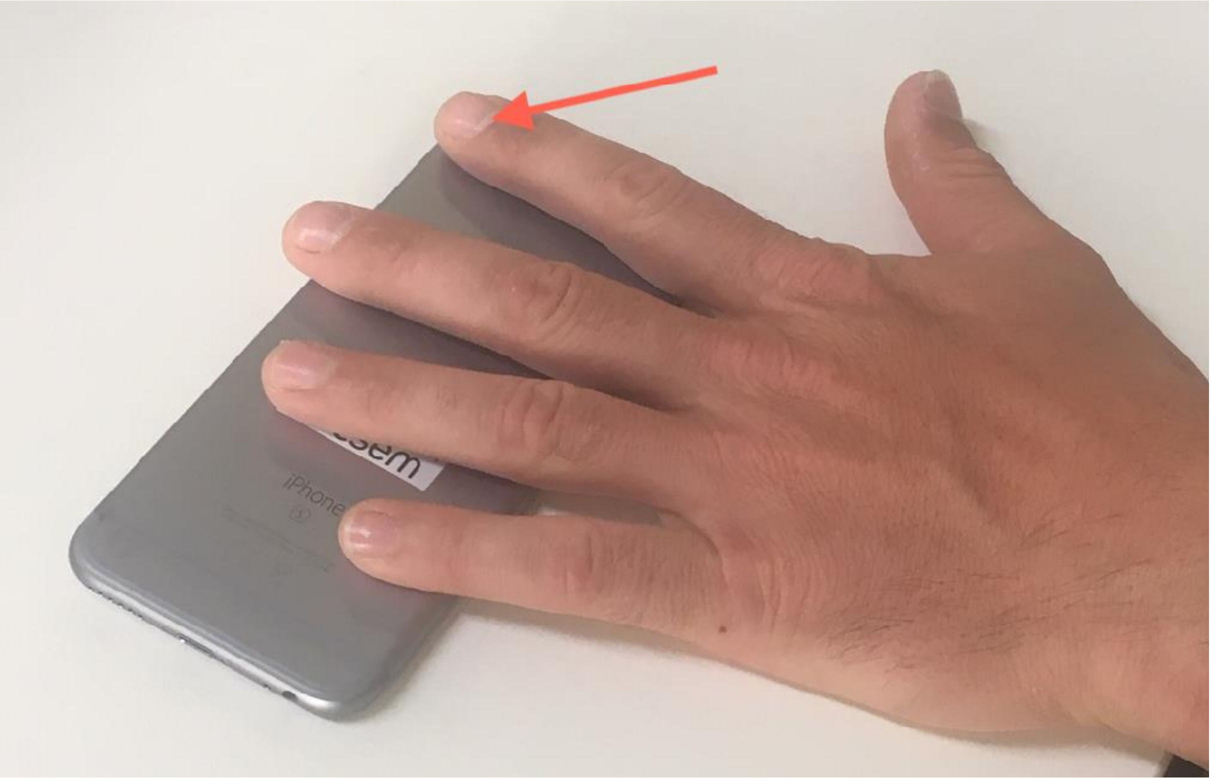

Supplemental Figure S1: Correct positioning of the patient's finger during the measurement. The arrow is pointing to the position of the camera.
